# Habituation to Transport Helps Reducing Stress-Related Behavior in Donkeys During Loading

**DOI:** 10.3389/fvets.2020.593138

**Published:** 2020-12-03

**Authors:** Francesca Dai, Silvia Mazzola, Simona Cannas, Eugenio Ugo Luigi Heinzl, Barbara Padalino, Michela Minero, Emanuela Dalla Costa

**Affiliations:** ^1^Dipartimento di Medicina Veterinaria, Università degli Studi di Milano, Milan, Italy; ^2^Direzione Sicurezza, Sostenibilità e Ambiente, Università degli Studi di Milano, Milan, Italy; ^3^Department of Agricultural and Food Science, University of Bologna, Bologna, Italy

**Keywords:** habituation, stress, behavior, welfare, transport, donkey

## Abstract

Adopting proper animal management strategies, including training, might reduce to a substantial extent the adverse effects of transport-related stress in animals. The aim of this study was to evaluate the effect of habituation to transport on stress-related behaviors and physiological indicators during loading and unloading in donkeys. Fourteen donkeys were recruited and divided in two treatment groups: Habituation (H; M = 5, F = 2) and Control (C; M = 5, F = 2). H donkeys were gradually habituated to be transported, traveling together with their mothers and other adult donkeys well-accustomed to transport, while C donkeys had never been transported before. Loading and unloading phases were video recorded and behavior was analyzed. Saliva samples for cortisol concentration determination were collected at rest and after unloading. Latency time to load was significantly shorter for H donkeys than C donkeys (Mann-Whitney; *p* = 0.004). C donkeys also showed significantly more stress-related behaviors (Mann-Whitney; *p* = 0.026) and required a higher but not statistically significant number of human interventions to load. Cortisol concentration increased in both groups, but no differences were found between them (Mann-Whitney; *p* > 0.05). These results suggest that habituation to transport could mitigate stress during loading procedures in donkeys reducing loading time, frequency of stress-related behaviors and diminishing the need of human intervention.

## Introduction

Throughout Europe, a population of about 395,910 donkeys is estimated (1), of which 93,468 are registered in the Equine Italian Database (2). Donkeys in Italy can be kept as pets, or used for leisure activities, therapy programs, or milk and meat production (3).

Transport is part of the management for the majority of pets and farm animals, including donkeys, having different purposes, such as reaching slaughterhouse, moving to a different farm, breeding, competitions and fairs, and medical procedures (4). Transport procedures are known to be stressful for animals, having both short term and prolonged effects on their welfare (4–7). It is also known that transport-related stress could influence meat quality in a variety of species (5, 8–10), thus potentially reducing profits derived from animal farming for meat production. When transported, several potential stressors can impact animal welfare, including interaction with humans, loading, unloading, and penning in a new, unfamiliar environment, and confinement with and without motion, vibrations, changes in temperature and humidity, inadequate ventilation, and often, deprivation of food, and water (11). In particular, loading is considered to be one of the most stressful components of transport for most animals, including equines (12–17), and it is reported to be the phase with the higher number of transport-related injuries particularly in horses which show transport related behavioral problems or have been trained with inappropriate training methods (18, 19). Several stressors are involved in pre-loading and loading procedures: separation from a familiar environment and social group (6), interactions with humans (14, 20), walking on the ramp (21), entering the trailer (22). Stress and fear during loading are also reinforced by recollections of previous unpleasant traveling experiences (11, 23–25).

The reduction of adverse effects of pre-transport factors decreases the probability of compromising animal welfare during the transport phase itself (7). The pre-transport preparation of animals plays an important role: it is reported that adopting proper management measures might reduce to a substantial extent the adverse effects of loading on stress, improving animal welfare (7). Habituation, in particular, is known to lead to decreased behavioral reactions to a previous novel situation (26), and habituation to loading as a foal, a yearling or an adult horse was reported to make loading behavior become as normal as walking into a stall (23). As highlighted by Padalino and Riley, people involved in equine transport should apply best practices, such as training of animals using evidence-based methodologies (4). In order to evaluate the efficacy of transport training methods, transport-related stress should be evaluated using both behavioral and physiological indicators. In horses, stress-related behaviors during loading include pawing, kicking out, bolting, head-shaking, and avoidance reactions, such as rearing, pulling away sideways, or backwards (13, 16, 17, 26, 27). During transport, reported stress-related behaviors are vocalizing, head tossing, pawing, scrambling, head-turning, kicking at the vehicle, biting and kicking at other horses, and reduced feeding/drinking (27–31). Reported unloading stress-related behaviors are a reluctance to exit the vehicle, prolonged immobility, and running off (21). To the authors' knowledge, no research has been conducted on donkeys to assess stress-related behavior during transport. Several physiological indicators have been proposed to evaluate transport related stress, both in horses and in donkeys, such as cortisol (11, 32–36), ß-endorphin (11, 36, 37), adrenocorticotropic hormone (32) and chromogranin-A (38), and infrared thermography (39).

The aim of this study was to evaluate the effect of habituation to transport procedures on stress related to loading and unloading, using behavioral indicators and salivary cortisol level, in donkeys.

## Materials and Methods

### Ethics Statement

This was an opportunistic study: no animals were transported to record data for the purposes of this study, no farm routine management has been modified for the purpose of the study. To obtain the best from each pasture, donkeys were routinely moved from one pasture to another. Therefore, no extra work was required to the farmer. No animals underwent more than a minimal distress. Transports were conducted in compliance with Council Regulation (EC) No 1/2005 of 22 December 2004 on the protection of animals during transport and related operations. Verbal informed consent was gained from the farmer prior to taking part in this research. Written consent was deemed unnecessary as no personal details of the participants were recorded.

### Animals and Facility

All the donkeys kept on farm were health checked and handled daily by the farm manager and/or the groom, so they were used to human contact. Fourteen healthy Romagnolo donkey foals (M = 10; F = 4; 1.2 ± 0.4 years) intended for meat production were included in the study. Only healthy foals born in 2018 were included in the study. Donkeys were born and raised on the same farm located in Northern Italy. Animals were group-housed at pasture with access to clean water *ad libitum* with both automatic drinkers and buckets. Donkeys were free to graze; pastures were managed to guarantee adequate nutrition in terms of quantity and quality of grass. If needed, depending on the season, weather, pasture conditions, and donkey growth rate, hay and mixed feed were provided.

### Treatments

Donkeys were randomly divided in two sex-balanced groups of seven subjects each: Control (C; M = 5, F = 2) and Habituation (H; M = 5, F = 2). All the animals were used to be handled and cared for by the same handlers. Foals in the H group were gradually habituated to be transported over short distances (from one pasture to another, about 30 min journeys), traveling together with adult donkeys with travel experience, including their own mothers. This habituation training started when the donkeys aged 6 months and lasted until they were taken to the slaughterhouse (1.2 ± 0.4 years). During the habituation, transport procedures were always performed using the same truck and by the same stockmen people, familiar to the donkeys (farm manager and groom). Foals were left free to load following other donkeys, taking advantage of their gregarious behavior, so they were not led by handlers or pushed by them in anyway. Donkeys in the H group were subjected to a minimum of 5 transports and no injuries were reported in both donkeys and donkey handlers. Donkeys in the C group were naïve to transport since they were housed together in a pasture, different from one of the H group, from birth. All the animals were used to the handlers: while donkeys in the C group were not used to be loaded nor to travel, animals were used to be handled and cared for by the same handlers.

### Data Collection

Data were collected during the transport from the pasture (where they were kept) to the main farm. For C donkeys, this was the first transport of their life. All donkeys involved in the study were transported in small groups (two to four donkeys, coming from the same familiar group) with the same truck, for a total of six transports. The transports started at around 4.30 p.m., and their durations ranged from 50 to 88 min (mean 64.69 ± 14.57 min). All transport procedures (loading and unloading) were performed by the stockmen according to the usual farm routine. Donkeys were conducted with a lead rope and gently encouraged to move by handlers, also offering food. Animals were loaded in group (two to four donkeys at a time) in order to take advantage of their gregarious behavior. At arrival, the truck door was opened, and the donkeys were left free to unload without leading or encouraging them.

### Behavioral Analysis

The loading and unloading phases were video recorded using an HD digital video camera (Canon Legria HFR88) controlled by the researcher. The loading time (from the procedures beginning, with the donkey being in front of the ramp, until the donkey had all four feet on the trailer) and time to unload (from the trailer doors opening until the donkey had all four feet on the ground) was directly recorded using a stopwatch. Donkey behavior during loading and unloading was separately analyzed by a treatment-blind animal scientist, experienced in equine behavior analysis. A focal animal continuous recording method was applied, using the software Solomon Coder beta 17.03.22. The frequency and duration of different behaviors were recorded. Since no literature is available on donkey behavior during loading and unloading, the ethogram was adapted from the one used for horses by Dai et al. (13) (Table 1).

**Table 1:** Ethogram for the evaluation of donkey behavior during loading and unloading [modified from (13)].

**Behavior**	**Description**	**Category**
Walk	The donkey walks toward the trailer	Forward locomotion
Trot	The donkey trots toward the trailer	Forward locomotion
Gallop	The donkey gallops toward the trailer	Forward locomotion
Backwards	The donkey moves away from the trailer	Stress-related behavior
Standing	The donkey stands on the four legs	Stress-related behavior
Turn back	The donkey tries to turn all its body in the opposite direction of the trailer	Stress-related behavior
Refuse to walk	The donkey stops moving, digging in its heels, refusing to proceed	Stress-related behavior
Rear	The donkey rears with its front legs	Stress-related behavior
Kick	The donkey kicks, one or two legs is lifted and moved rapidly and forcefully	Stress-related behavior
Mount	The donkey mounts the donkey in front of him/her	Stress-related behavior
Defecate	The donkey drops manure	Stress-related behavior
Urinate^*^	The donkey drops urines	Stress-related behavior
Paw^*^	The donkey rises a foreleg and scrapes the floor	Stress-related behavior
Sniffing^*^	The donkey sniffs the ground	Stress-related behavior

* *Behaviors that were not observed were not considered for the statistical analysis*.

Furthermore, each interaction between the handlers and the donkeys was noted from videos. Any interactions to facilitate loading was considered (pulling the rope, pushing the donkey from the back, inciting the animal, offering food).

### Salivary Cortisol Evaluation

For cortisol concentration determination, saliva samples were collected using SalivaBio Children's Swab (Salimetrics®, Carlsbad, CA, USA) in the pasture with donkeys at rest immediately before starting loading procedures and immediately after unloading. In order to minimize the impact of the circadian pulsatile cortisol release pattern (40), for each donkey two more samples were taken under control conditions (at the pasture), on the days immediately prior to transport, in the same time slot in which the transport was scheduled (between 4 p.m. and 5 p.m.). The swab was inserted in donkey's mouth, gently restraining the animal with a head collar; the donkey was left free to chew the swab for 1–2 min, then the swab was put in the device tube, closed with a plastic stopper to prevent evaporation, placed in ice and then stored at −20°C immediately after it arrived at the laboratory. The temperature was maintained until analysis. At the time of analysis, the samples were thawed at room temperature and centrifuged (3,500 rpm for 15 min, at 4°C) according to the protocol for salivary samples. Analysis was performed using a commercially available multispecies cortisol enzyme-linked immunosorbent assay (ELISA) kit (Enzo Life Sciences, Farmingdale, NY, USA), following previously validated protocols (41). Samples were aliquoted into wells in duplicate (100 μL), and absorbance measured using a wavelength of 405 nm in a microplate plate reader (Multiskan EX, LabSystem, Thermo Fisher Scientific, Milan, Italy). A recovery test was applied to determine if the value obtained from our samples were accurate (e.g., no interferences with the measurements due to the presence of undesired factors in the sample matrix). The mean recovery was 109.1% ± 8.4, while the average intra- and inter-assay coefficients of variation, respectively, were 3.9 and 7.8%. The assay sensitivity was 56.72 pg/ml (range 156–10,000 pg/ml). The laboratory researcher was blinded to the hypotheses and conditions.

### Statistical Analysis

Behaviors of the categories forward locomotion and stress-related behaviors were considered together for the statistical analysis (Table 1). Based on the total length of the observation of the video recordings, durations of behaviors were calculated as percentage of total observation time (proportional duration time). Behaviors that were not observed (urinate, paw, sniffing) were not considered for the statistical analysis. Cortisol variations (delta) for each subject of the two groups were calculated. Statistical analysis was performed using SPSS 25 (SPSS Inc., Chicago, IL, USA). Data were tested for normality and homogeneity of variance using the Kolmogorov-Smirnov and Levene test, respectively. Mann-Whitney test was used to investigate differences between groups in behavior during loading and unloading, time to load and unload, human intervention, and cortisol concentration (delta). Statistical significance was accepted at *p* ≤ 0.05.

## Results and Discussion

### Behavior Analysis

**Figure 1:**
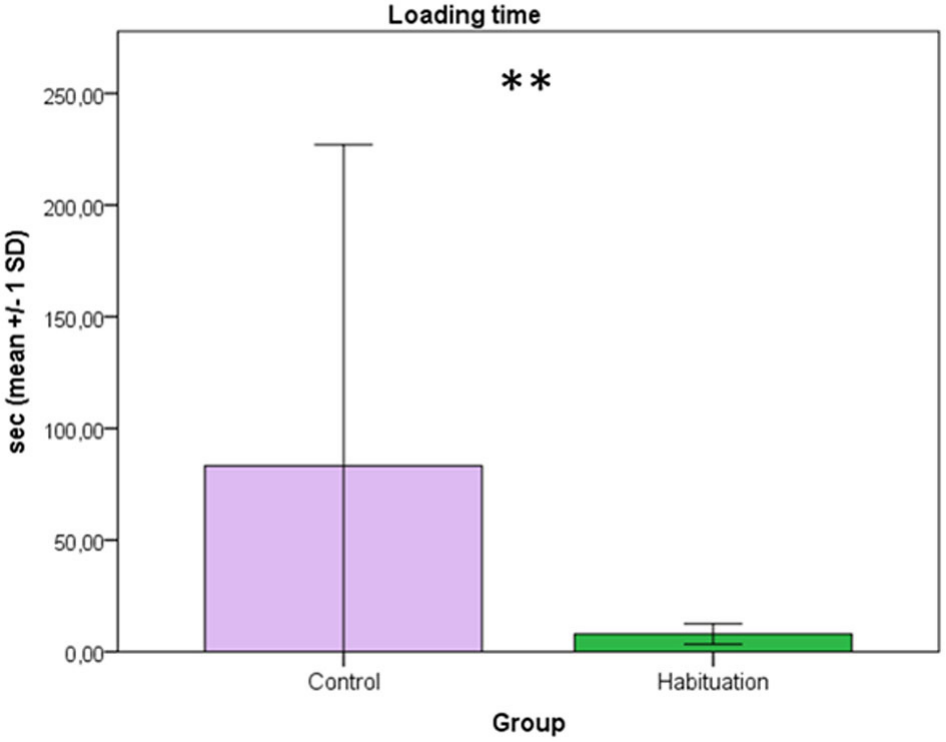
The time (sec) for loading is presented on the y-axis (mean ± 1 SD) with the groups (control vs. habituation) on the x-axis (Mann-Whitney test; **p = 0.004).

**Figure 2:**
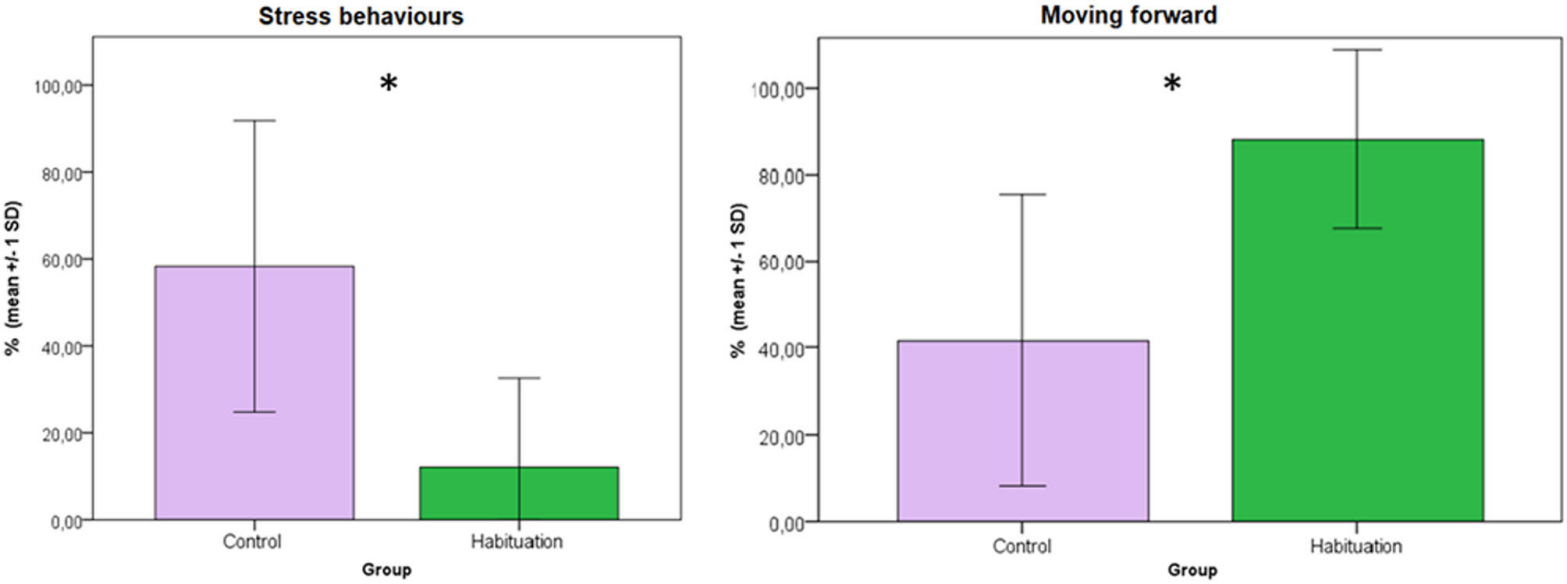
Mean percentages of time (±1 SD) of stress related and moving forward behaviors of donkeys in the two treatment groups during loading procedure. (Mann-Whitney test; *p < 0.05).

Results of the behavioral analysis showed that latency time to load was significantly shorter in H donkeys (mean 7.97 ± 4.62 s) than in C donkeys (mean 83.23 ± 143.84 s) (Mann-Whitney test; *p* = 0.004) (Figure 1). H donkeys showed more forward locomotion toward the truck than C donkeys (87.89 ± 20.48% and 41.71 ± 33.51%, respectively; Mann-Whitney test; *p* = 0.026). Furthermore, C donkeys showed significantly more stress-related behaviors than H donkeys (58.29 ± 33.51% and 12.11 ± 20.48%, respectively; Mann-Whitney test; *p* = 0.026) (Figure 2). These results are similar to those reported in trained horses (13, 19). However, positive and negative training reinforcements might require more time than farmers will dedicate (13); for this reason, the proposed and tested habituation training, including the foal following the mother and other known conspecifics, seems instead to be effective in donkeys and may prove to be more feasible when introduced in an on-farm routine as the trailer could be left in the pasture so that the animals can explore it and get habituated to load and unload. Habituation has been strongly recommended for horses (22) and was proven to minimize the incidence of transport related behavioral problems and subsequently injuries (18). In the latter study, self-loading also was found associated with a reduction of loading problem behavior and injuries, however, it is worth to know that self-loading require lots of time, effort, and training skills. Even though, habituation requires some time, Houpt et al. (22) clearly tested that when a foal is habituated to load into a trailer following the mare, loading into a trailer becomes easy as walking into a box for both the foal and its handler. This is the first study where this training to transport procedures using habituation with the foal following conspecifics was tested in donkeys.

Group C donkeys required a higher but not statistically significant number of human interventions to load compared to H's (H: mean 1.29 ± 0.95; C mean: 7.43 ± 14.03, Mann-Whitney test; *p* = 0.32). The lack of statistically significant difference may probably be due to the high individual variability observed in C subjects and it needs to be ascertained with further studies. However, it is worth noting that this result has interesting practical fallouts considering both animal welfare and human safety. Indeed, interactions with large animals may become dangerous for handlers, especially when animals are stressed and/or frightened: several studies conducted in sport horses with behavioral problems related to transport highlighted the high occurrence of injuries in humans during loading, such as rope burns, lost fingers, broken bones, or bruises, and bleeding (17, 18, 42). Not only loading may be risky for the handlers, but inappropriate animal management in this transport phase has been reported to be a risk factor also for horse injuries (19, 42). The adoption of an adequate training for loading has been deemed useful in increasing human safety by reducing horse-related injuries among handlers (18).

Even if loading represents the most stressful stage of animal transport (7, 12, 43), also unloading may be challenging. Critical factors are steepness and slipperiness of the ramp, and the novel environment the animals are required to enter (6). Consequently to stress and/or anxiety related to unloading, horses have been observed freezing inside the vehicle or performing flight responses (21). In the present study, donkeys did not exhibit abnormal behavior during the unloading phase, and no differences between groups were found in the unloading time (Mann-Whitney test; *p* > 0.05), with C group unloading in 48.9 ± 32.4 s (mean ± 1 SD), while H group unloading in 71.0 ± 31.2 s. Besides, the behavior of the donkeys in the two groups was similar during the unloading procedure (Mann-Whitney test; *p* > 0.05). Having traveled with other members of the social group could have contributed to attenuating the stress at the time of unloading. Taking advantage of the donkeys' gregarious attitude, the animals got out of the truck without showing behaviors attributable to stress.

### Salivary Cortisol Evaluation

**Figure 3:**
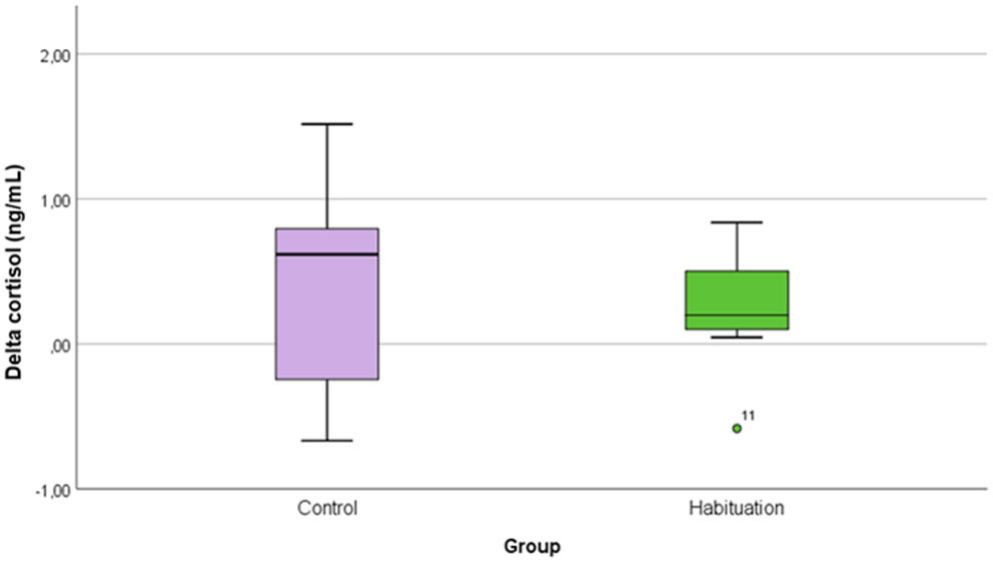
Box plot of the transport-related salivary cortisol variation (delta) in the two groups of donkeys. Outliers are represented with dots.

No differences were found in delta cortisol concentration between groups (Mann-Whitney test; *p* > 0.05) (Figure 3). From Figure 3, it is evident the great variability of group C data, much greater than those of the group H. In case of acute stress, cortisol secretion increases significantly, with a secretion level that varies from individual to individual, depending on the individual perception of the stressor, but described as correlated with the intensity of the stress (44). These results highlighted the subjectivity of the activation of the hypothalamic-pituitary-adrenal cortex axis induced by the stressogenic stimulus: this variability, associated with the small number of donkeys, may be the basis of the lack of significance of the data. The great variability may be also related to the different space allowance and conditions (group of 2 or 4) during the different journeys tested.

### Limitations and Future Perspectives

In this study, no donkeys were transported only for the purpose of data collection: all the transports were part of the farm's management procedures. The limited size of the farm has led to a small number of donkeys available. In a convenient geographical location, there were no donkey farms with the same donkey breed with similar management: therefore, to avoid data bias (especially considering physiological data), the number of subjects observed was not integrated with those of other farms. It clearly appears that the sample size and the unique provenience of the evaluated donkeys represent limitations for the generalization of our results. Further studies applying training to transport procedures through habituation with the foal following conspecifics are foreseen to generalize the results to donkeys kept for other purposes and subjected to different management. As stress related to transport could be affected by several potential stressors (11), future studies should also consider to evaluate different habituation protocols such as load on a trailer without movement and habituation to loading on a trailer and the vehicle movement.

Donkeys were not habituated to saliva sampling. Even if the method is reported to be non-invasive and should not induce a significant stress to the animal, being rapid and permitting animal mobility (38, 41), the procedure represented a novel stimulus for the animal. Although the time required for sampling was not sufficient to allow the presence of free cortisol in the saliva (45), it may not be excluded that the procedure induced a certain degree of stress in donkeys, therefore influencing our result. However, the applied methodology was the same for H and C groups, eliminating any potential bias between treatment groups. Cortisol is released from the adrenal glands in pulses controlled by the hypothalamus's paraventricular nucleus, which receives circadian pulses from the suprachiasmatic nucleus of the hypothalamus and integrates information from cognitive processes and emotional and physical stress reactions (46). The cortisol secretory pulses' variations result from the ultradian rhythm: the secretory episodes occur at a relatively stable frequency, with variable amplitudes, responsible for the typical circadian rhythm. Over 24 h, between 15 and 22 secretory cortisol pulses are expected, with an early morning peak and a nadir by the first half of the night. In the present work, we tried to minimize the impact of this pulsatile circadian release of cortisol: all transport took place in the afternoon, and, for each animal, salivary sampling was at the transport and also the day before, the same time as transport was planned.

Measuring other physiological indicators, such as heart rate variability (HRV), respiratory parameters, beta-endorphin, catecholamines or glucose levels, would have increased the scientific robustness of results, however, it would have also decreased the study's feasibility increasing the invasiveness of the data collection.

As one of the reasons for breeding donkeys is meat production and numerous studies highlighted how transport stress negatively affects meat quality in several species [see (5) for review]. In future studies, it would be interesting to analyze the effect of transport related stress on donkey meat. As a matter of fact, only few studies described the incidence of transport related stress on equine meat quality (47, 48).

Regardless of the above-mentioned limitations, to the authors' knowledge, this is the first study documenting the effects of habituation to transport procedures in donkeys. In the present study, meat donkeys were taken only as a model, as two groups of animals of the same breed, balanced for sex and age, with the same management and handling from the same stockmen could be subject to the different training procedure. As donkeys are frequently transported for several purposes, including changing ownership, leisure activities, therapy, sport activities, habituation following conspecifics could be helpful in reducing stress related to transport. This result has a practical fallout, since habituation with the foal following the conspecifics could be more feasible, easier and should be recommended for donkeys.

## Conclusions

These results, although preliminary, suggest that habituation to transport following conspecifics could mitigate stress responses during loading in donkeys, reducing loading time, the frequency of stress-related behaviors and the handler's intervention. Further research, conducted on a larger donkey population on several farms, is needed in order to confirm these results.

## Data Availability Statement

The raw data supporting the conclusions of this article will be made available by the authors, without undue reservation.

## Author Contributions

FD, ED, and MM: conceptualization. FD and BP: methodology. ED: formal analysis and visualization. FD, EH, and SM: investigation. MM: resources. FD: data curation. FD, SM, and ED: writing—original draft preparation. MM, SC, ED, EH, and BP: writing—review and editing. MM: supervision and project administration. All authors have read and agreed to the published version of the manuscript.

## Conflict of Interest

The authors declare that the research was conducted in the absence of any commercial or financial relationships that could be construed as a potential conflict of interest.
